# Psychosis with untreated hypothyroidism: How a generalist made a difference

**DOI:** 10.51866/cr.897

**Published:** 2026-01-10

**Authors:** Shahrizam Tahir, Nur Suhaila Idris, Aida Maziha Zainudin

**Affiliations:** 1 Department of Family Medicine, Universiti Sains Malaysia, Kota Bharu, Kelantan, Malaysia.; 2 Department of Pharmacology, Universiti Sains Malaysia, Kota Bharu, Kelantan, Malaysia.

**Keywords:** Hypothyroidism, Primary healthcare, Psychotic disorder, Myxedema, Interdisciplinary communication

## Abstract

Myxoedema psychosis is a rare but treatable condition resulting from severe hypothyroidism. We report the case of a man who presented to a primary healthcare facility seeking a medical report for financial assistance, having lost his job after being treated for schizophrenia. A comprehensive re-evaluation raised the possibility of endocrinopathy-induced psychosis as one of the differentials. The diagnostic process was challenging due to the rarity of the condition and the absence of classic hypothyroid features. Laboratory investigations revealed markedly low free thyroxine levels and elevated thyroid-stimulating hormone and anti-thyroid peroxidase antibody levels, confirming the diagnosis. Treatment with levothyroxine led to significant clinical improvement. This case underscores the vital role of primary care providers in recognising organic causes of psychosis through patient-centred assessment, application of primary care principles and interdisciplinary collaboration. It also highlights the potential consequences of misdiagnosis in atypical psychiatric presentations, including functional decline and job loss, and reinforces that appropriate management can lead to optimal outcomes.

## Introduction

Primary hypothyroidism is characterised by reduced circulating free thyroxine levels and elevated thyroid-stimulating hormone (TSH) levels.^1^ The presence of increased anti-thyroid peroxidase (anti-TPO) antibody levels suggests an autoimmune aetiology, consistent with Hashimoto thyroiditis.^2^ The prevalence of hypothyroidism ranges from 0.5% to 18%, depending on the population studied.^3^ According to a systematic review, 17.3% of hypothyroidism cases are due to Hashimoto thyroiditis.^4^ Hypothyroidism is commonly associated with dyslipidaemia, including high cholesterol, triglyceride and low-density lipoprotein levels.^4,5^ This adverse cardiovascular impact should alarm clinicians to the danger of long-standing, untreated hypothyroidism, warranting early detection and treatment.

Commonly, hypothyroidism presents as fatigue, lethargy, cold intolerance, weight gain, constipation and coarse hair.^6^ Though infrequent, neuropsychiatric symptoms such as altered mental status and psychosis have been linked to hypothyroidism.^7,8^ The term ‘myxoedema madness’ was first used in 1949, describing psychosis in patients with severe hypothyroidism.^9^ The exact pathophysiology remains unclear^10^; however, animal studies have suggested that thyroid hormone receptors in the limbic system play a critical role in emotional and behavioural regulation. Thyroid hormone dysregulation is believed to contribute to this condition.^11^ In humans, the reduced availability or impaired transport of triiodothyronine within the central nervous system has resulted in reduced neuronal activities reflected in psychiatric symptoms.^3^ The estimated prevalence of psychosis in hypothyroidism reported in contemporary studies is below 5%, with the most common form being the delusion of persecution.^12^

Hypothyroidism often co-exists with schizophrenia; a South Korean study reported a prevalence of 4.9%, while a US study demonstrated an association with an odds ratio of 1.88.^13^ This overlap complicates diagnosis, as myxoedema psychosis often presents with non-specific psychiatric symptoms such as delusions, while classic physical signs may be absent.^4,10^ Some patients require both antipsychotics and thyroid hormone replacement, whereas others show clinical improvement with L-thyroxine alone.^4,14^ The literature reveals an excellent prognosis, with complete remission achieved in 82.7% of cases. These findings underscore the importance of screening for hypothyroidism in patients presenting with psychosis.^4^

In our case, the patient presented with disorganised behaviour, persecutory delusions and a history of aggressive behaviour. These non-thyroid-specific symptoms pose diagnostic challenges, which could lead to misdiagnosis and delayed treatment.

## Case presentation

A 39-year-old man visited a tertiary hospital primary care clinic requesting a medical report to obtain financial aid from the Department of Social Welfare. He had a history of hospitalisation in the centre 9 months prior due to alleged physical aggression and a 6-month duration of continuous disturbance in the form of fearfulness, suspiciousness and disorganised behaviour.

Assessment during his hospital admission showed negative results for syphilis, human immunodeficiency virus and hepatitis B and C. Urine toxicology tests yielded negative findings. Renal tests revealed chronic kidney disease (CKD) stage 3B, elevated liver enzyme levels and hypercholesterolaemia. A non-contrast computed tomography (CT) scan of the brain showed unremarkable findings. Liver and renal ultrasounds revealed fatty liver and renal parenchymal changes. However, there were no documented thyroid function test (TFT) results during the initial hospital admission (Table 1). The patient was prescribed amisulpride 200 mg twice daily for schizophrenia. However, after discharge, he did not return for psychiatric follow-ups and had not been taking his medication as instructed.

**Table 1:** Investigation summary during admission and clinic assessment.

Test	February 2024	October 2024	Normal range
Free T4	Not available	<0.5 pmol/L	12–22 pmol/L
TSH	Not available	>100 mlU/L	0.270–4.20 mlU
Anti-TPO	Not available	2165 klU/L	≤34 klU/L
TRAb	Not available	943 klU/L	≤115 klU/L
WCC	5.84×10^9^	6.19×10^9^	3.8-9.7×10^9^
Hb	12.4 g/dL	12.9 g/dL	13.5-17.4 g/dL
MCV	99.0 fL	94.8 fL	78.9-95.7 fL
MCH	31.4 pg	30.7 pg	25.4-31.1 pg
PLT	212×10^9^	253×10^9^	167-376
CRP	<0.6 mg/L	<0.8 mg/L	<5 mg/L
FBS	4.3 mmol/L	5.0 mmol/L	<6.1 mmol/L
HbAlc	5.2%	Not repeated	<5.7%
Na	136 mmol/L	137 mmol/L	136-145 mmol/L
K	4.6 mmol/L	4.1 mmol/L	3.5–5.1 mmol/L
Urea	5.8 mmol/L	5.7 mmol/L	2.76-8.07 mmol/L
Creatinine	186 mmol/L	159 μmol/L	62-106 μmol/L
eGFR	38 (Stage 3B)	46 (Stage 3A)	
ALT	275	193 U/L	10-50 U/L
ALP	80	No reagent	
AST	256	244	10-50 U/L
Alb	46	No reagent	
TBil	9	10 μmol/L	<24 μmol/L
DBil	4	4 μmol/L	<4.3 μmol/L
Chol	8.97 mmol/L	9.02 mmol/L	<5.18 mmol/L
HDL	2.65 mmol/L	2.30 mmol/L	According to risk
LDL	5.66 mmol/L	6.3 mmol/L	
Triglyceride	1.02 mmol/L	1.02 mmol/L	<1.7 mmol/L
Urine protein	Trace		
Urine culture	No growth		
HBsAg	Non-reactive	Not repeated	
HIV Ab/Ag	Non-reactive		
Syphilis	Non-reactive		
Brain CT	The brain CT findings were normal. There was neither ischaemia, haemorrhage nor mass. The ventricular size was normal, and no midline shift was noted.		
USG KUB	Features of renal parenchymal disease were observed, with preserved bilateral kidney size and cortical thickness. There was no focal lesion, calculus or hydronephrosis.		
USG HBS	The liver echogenicity was increased, with a smooth liver margin. The liver span was normal. Neither focal lesion nor biliary tree dilatation was found. The gallbladder was well distended, with no calculus within.		

TSH: thyroid-stimulating hormone, anti-TPO: anti-thyroid peroxidase, TRAb: thyrotropin receptor antibody, WCC: white cell count, Hb: haemoglobin, MCV: mean corpuscular volume, MCH: mean corpuscular haemoglobin, PLT: platelet, CRP: C-reactive protein, FBS: fasting blood sugar, HbAlc: haemoglobin A1c, Na: sodium, K: potassium, eGFR: estimated glomerular filtration rate, ALT: alanine transaminase, ALP: alkaline phosphatase, AST: aspartate aminotransferase, Alb: albumin, TBil: total bilirubin, DBil: direct bilirubin, Chol: total cholesterol, HDL: high-density lipoprotein, LDL: low-density lipoprotein, HBsAg: hepatitis B surface antigen, HIV Ab/Ag: human immunodeficiency virus antibody/antigen, CT: computed tomography, USG: ultrasound, KUB: kidney, ureter and bladder, HBS: hepato-biliary system.

Upon further evaluation, the patient experienced psychotic symptoms, including delusions of persecution, believing a satellite was monitoring him, and someone was plotting to kill him and his family. He also experienced a delusion of reference, stating that the television was talking about him. His family reported that he sometimes talked to himself, pacing at home, taking frequent showers and not drying himself properly afterwards. He denied having visual or auditory hallucinations, thought alienation and passivity phenomena. The interview revealed no mood symptoms or any changes in appetite. The triggering factors identified included a recent newborn son, causing additional responsibility and a major change in his daily routine. Additionally, he reported increased fatigue. These symptoms collectively impaired his ability to work. Job loss due to a health condition prompted him to request a medical report for financial aid. Otherwise, there were no other classical symptoms of hypothyroidism, such as weight gain, cold intolerance, constipation or dry skin. He did not have any aggressive tendencies.

The patient was married, with four children, and served as the family’s sole breadwinner. He was previously employed as a restaurant waiter. He and his family did not own a vehicle and relied on public transportation for hospital visits. They were residing in a suburban area approximately 30 km from the tertiary hospital. His wife and older brother were identified as his primary sources of social support. There was neither a family history of mental illness nor any personal history of illicit drug use.

On examination, his vital signs were within normal range. His body mass index was 27 kg/m^2^. Cardiovascular examination revealed no clinical signs of heart failure. The jugular venous pressure was not elevated; the apex beat was not displaced; and on auscultation, heart sounds were normal, with no added sounds or murmurs. He was oriented to time, place and person but appeared blunted, avoided eye contact and frequently walked away during the interview. His speech was of low volume, monotonous, at a slower speed and occasionally irrelevant. He denied hallucinations. Insight was poor, with paranoid thoughts. He reported his mood as okay; however, his affect was restricted. He had dry skin and a delayed relaxation phase of reflex. Neck examination revealed no swelling or scarring. There was no pedal oedema or voice changes, and other neurological findings were normal.

Electrocardiogram revealed a normal sinus rhythm, a heart rate of 75 beats per minute and a QTc interval of 390 ms. Tracing showed preserved QRS amplitudes, appropriate R-wave progression across precordial leads and no evidence of ventricular strain patterns or ischaemic changes. Additionally, his full blood count, electrolyte levels and diabetic screening results were normal. Given his condition, TFT, TRAb and anti-TPO screening were conducted. High anti-TPO reading indicated Hashimoto thyroiditis.

Having been diagnosed with severe hypothyroidism secondary to Hashimoto thyroiditis, the patient was started on L-thyroxine 100 mcg daily (1.5 mcg/kg/day). Consultation for comanagement with an endocrinologist and a psychiatrist was sought. The psychiatrist planned to resume amisulpride and follow up in the clinic, while the endocrinologist continued L-thyroxine and scheduled a review in 6 weeks with repeat TFTs. The patient was jointly managed with a gastroenterologist. He was started on ezetimibe 10 mg daily for dyslipidaemia with transaminitis. The gastroenterologist planned to review his liver function test (LFT) trend pending secondary investigations. Measures to slow CKD progression were initiated while the patient’s renal profile was closely monitored in primary care.

After reporting clinical improvement with treatment, the patient did not return for follow-up. Multiple attempts were made to contact the patient and his immediate family members (i.e. wife and older brother) to remind them about the importance of follow-up. They cited geographical distance and transportation challenges as barriers. A referral was then facilitated to the nearest clinic for ongoing management to address these challenges. As levothyroxine was initiated alongside antipsychotic therapy and the patient was lost to follow-up, a definitive causal relationship between hypothyroidism and psychosis could not be established.

## Discussion

This case highlights the significance of applying primary care principles to effectively identify and manage rare diseases. As primary care providers encounter diverse cases across all specialities, it is vital to approach patients individually and holistically.^15^ By adopting a generalist approach, clinicians can view patients as a whole rather than as separate organs and diseases.^16^ In this case, a detailed re-evaluation of the patient’s clinical condition before finalising the medical report revealed a reversible cause of hypothyroidism-induced psychosis as one of the differentials. Given that hypothyroidism can co-exist with schizophrenia,^14^ primary care providers must maintain a broad differential diagnosis when assessing patients with worsening psychosis. The co-existence of schizophrenia and hypothyroidism poses clinical challenges. Untreated hypothyroidism can cause worsening of schizophrenia by exacerbating negative and cognitive symptoms, mimicking the psychiatric illness.^14^ This shows the importance of primary care providers in implementing the principle of family medicine in every patient encounter, regardless of the chief complaint.

The approach to psychosis requires a systematic evaluation to exclude organic causes. Medical conditions such as vitamin B12 deficiency, autoimmune disorders, drug toxicity and thyroid imbalances should be ruled out.^17^ They are treatable and, with timely intervention, can lead to effective recovery. The German Association for Psychiatry, Psychotherapy and Psychosomatics advises detailed history-taking, risk assessment for mental illness, comprehensive laboratory tests and brain imaging including either MRI or CT.^18^ This case demonstrates how such methods effectively identify organic causes of psychosis, enabling accurate diagnosis and successful treatment for optimal outcomes. Primary care providers who usually encounter patients first need to make a thorough clinical assessment and consider organic causes, such as myxoedema psychosis, while considering the co-existence of hypothyroidism and schizophrenia in unexplained psychosis.

Differentiating psychosis secondary to hypothyroidism from a co-morbid schizophrenia is clinically challenging due to overlapping symptoms.^8^ Myxoedema psychosis typically presents as a first psychotic episode in middle-aged or older adults with no psychiatric history and may be accompanied by confusion.^12^ In contrast, schizophrenia is a chronic disorder with an earlier onset.^19^ Both conditions may present with persecutory delusions and auditory hallucinations.^19^ Physical signs such as dry skin, facial oedema, voice hoarseness, bradycardia or delayed tendon reflex may suggest hypothyroidism.^2^ Myxoedema psychosis generally resolves with levothyroxine therapy, often permitting discontinuation of antipsychotics, whereas schizophrenia requires long-term antipsychotic treatment even after thyroid function has normalised.^13^

The approach to the treatment of psychosis depends on its aetiology. In myxoedema psychosis, reversing hypothyroidism by supplementing L-thyroxine results in significant improvement of symptoms. Weight-based L-thyroxine remains the drug of choice in hypothyroidism secondary to Hashimoto thyroiditis.^20^ The goal of treatment is to restore the state of euthyroid. A metaanalysis showed that correcting hypothyroidism led to clinical improvement in 82.3% of patients with myxoedema psychosis.^4^ The treatment of co-existing hypothyroidism and schizophrenia is more complex. Other than antipsychotics, patients require L-thyroxine supplementation, which necessitates close monitoring of thyroid function. Periodic monitoring is important, as some antipsychotics can suppress thyroid activity.^13^ Patients who maintain good adherence to both antipsychotic treatment and L-thyroxine replacement generally demonstrate favourable outcomes, with improvement in physical health and better control of psychotic symptoms.^13^

Primary care ensures continuity of care and facilitates coordination with different specialities for optimal outcomes. In this case, the concurrent prescription of levothyroxine and amisulpride improved the patient’s symptoms. The collaboration with psychiatric and endocrinology services ensured an interdisciplinary approach and optimised the patient’s outcomes. The gastroenterology subspecialty was consulted concerning his concurrent dyslipidaemia and transaminitis to ensure a holistic approach. Furthermore, adequate replacement of thyroid hormone normalised his TSH level and lipid profile, thus preventing detrimental cardiovascular disease.^20^ Once again, coordination of care, a principle in family medicine, proves to be a vital practice in bridging the gap between patient needs, psychiatric illness and endocrine specialities.

Although the patient’s main reason for visit was to seek financial assistance from the Department of Social Welfare, it remains essential for primary care providers to conduct a systematic and thorough evaluation while addressing the patient’s individual concerns. Endorsing financial assistance without proper assessment carries the risks of misclassification and may stigmatise a person with disability who has a potentially reversible medical condition.

Awareness of endocrine pathologies in mental illness is important. Thyroid disorders are among the seven ‘masquerades’ described in *Murtagh’s General Practice.* This case illustrates how severe hypothyroidism can present as primary psychosis, underscoring the importance of screening for endocrine dysfunction in psychiatric presentations. Such vigilance is consistent with Malaysian clinical practice guidelines and may help reduce diagnostic delays and healthcare system burden. We recommend that primary care providers maintain a high index of suspicion for thyroid dysfunction in patients presenting with psychotic symptoms, particularly in the absence of risk factors for schizophrenia, such as family history of psychotic illness, prior mental health problems or substance use disorder.

Loss to follow-up is a well-recognised challenge among patients receiving treatment for mental disorders. Contributing factors include low educational attainment, inadequate social support, limited mobility and poor or insufficient communication. Primary care providers must adopt proactive strategies to minimise this risk, such as maintaining consistent patient engagement, providing education on the importance of adherence and establishing dedicated teams to trace patients who default on care. The use of technology should also be optimised to support both clinicians and patients. In this case, continuity of care was facilitated through active tracing, direct communication with the patient and his family, appointment reminders and the mitigation of logistical barriers to follow-up. These efforts align with the core principles of family medicine, particularly continuity of care.^16^

## Conclusion

This case highlights the pivotal role of primary care providers in the early recognition and management of rare but treatable conditions such as myxoedema psychosis. Maintaining a broad differential diagnosis and considering organic causes, including thyroid dysfunction, in patients presenting with psychosis can prevent misdiagnosis and facilitate timely intervention. Overall, this report emphasises the importance of vigilance and interdisciplinary collaboration and the central role of primary care in bridging the interface between mental health and systemic disease.
